# A Novel, Multiple-Antigen Pneumococcal Vaccine Protects against Lethal *Streptococcus pneumoniae* Challenge

**DOI:** 10.1128/IAI.00846-18

**Published:** 2019-02-21

**Authors:** Win-Yan Chan, Claire Entwisle, Giuseppe Ercoli, Elise Ramos-Sevillano, Ann McIlgorm, Paola Cecchini, Christopher Bailey, Oliver Lam, Gail Whiting, Nicola Green, David Goldblatt, Jun X. Wheeler, Jeremy S. Brown

**Affiliations:** a Centre for Inflammation and Tissue Repair, UCL Respiratory, Division of Medicine, University College Medical School Rayne Institute, London, United Kingdom; b ImmunoBiology Ltd., Babraham, Cambridge, United Kingdom; c National Institute for Biological Standards and Control, South Mimms, Potters Bar, Hertfordshire, United Kingdom; d UCL Great Ormond Street Institute of Child Health, London, United Kingdom; Albert Einstein College of Medicine

**Keywords:** *Streptococcus pneumoniae*, multiple-antigen vaccine, protein antigen, vaccines

## Abstract

Current vaccination against Streptococcus pneumoniae uses vaccines based on capsular polysaccharides from selected serotypes and has led to nonvaccine serotype replacement disease. We have investigated an alternative serotype-independent approach, using multiple-antigen vaccines (MAV) prepared from S. pneumoniae TIGR4 lysates enriched for surface proteins by a chromatography step after culture under conditions that induce expression of heat shock proteins (Hsp; thought to be immune adjuvants). Proteomics and immunoblot analyses demonstrated that, compared to standard bacterial lysates, MAV was enriched with Hsps and contained several recognized protective protein antigens, including pneumococcal surface protein A (PspA) and pneumolysin (Ply). Vaccination of rodents with MAV induced robust antibody responses to multiple serotypes, including nonpneumococcal conjugate vaccine serotypes. Homologous and heterologous strains of S. pneumoniae were opsonized after incubation in sera from vaccinated rodents. In mouse models, active vaccination with MAV significantly protected against pneumonia, while passive transfer of rabbit serum from MAV-vaccinated rabbits significantly protected against sepsis caused by both homologous and heterologous S. pneumoniae strains. Direct comparison of MAV preparations made with or without the heat shock step showed no clear differences in protein antigen content and antigenicity, suggesting that the chromatography step rather than Hsp induction improved MAV antigenicity. Overall, these data suggest that the MAV approach may provide serotype-independent protection against S. pneumoniae.

## Abstract (precis)

Current vaccination against Streptococcus pneumoniae uses vaccines based on capsular polysaccharides from selected serotypes and has led to nonvaccine serotype replacement disease. We have investigated an alternative serotype-independent approach, using multiple-antigen vaccines (MAV) prepared from S. pneumoniae TIGR4 lysates enriched for surface proteins by a chromatography step after culture under conditions that induce expression of heat shock proteins (Hsp; thought to be immune adjuvants).

## INTRODUCTION

Streptococcus pneumoniae is a common cause of community-acquired pneumonia (CAP), septicemia, and meningitis (1), as well as of noninvasive diseases, such as acute otitis media (AOM) and bronchitis (2). Over 90 different serotypes of S. pneumoniae, determined by the characteristics of the capsular polysaccharide (CPS), have been identified (3). There are currently two vaccines available to prevent S. pneumoniae infections: the pneumococcal polysaccharide vaccine (PPV) and the pneumococcal conjugate vaccine (PCV). Each consists of capsular polysaccharide antigen from a limited panel of S. pneumoniae serotypes. In the United Kingdom, PPV remains the first choice for adult vaccination (4), and PCV is routinely included in childhood immunization schedules worldwide, as it has greater efficacy than PPV in infants. Unfortunately, in developing countries the high cost of PCV restricts its availability, and in addition, serotype coverage is reduced, as PCV was designed to include the most prevalent serotypes in North America (5). Furthermore, serotype replacement in response to PCV vaccination alters the ecology of S. pneumoniae, reducing the efficacy of polysaccharide vaccines over time (6). A vaccine based on protein antigens may provide a low-cost alternative approach capable of inducing cross-serotype protection (7, 8).

One vaccine approach dependent on protein antigens is a whole-cell approach, a cost-effective method of immunizing with a large number of potential protein antigens to potentially induce serotype-independent protective immunity. In addition, a whole-cell approach could target both humoral and cellular host immunity (9, 10), potentially enabling clearance of both disease and colonization. Several groups have therefore studied a whole-cell vaccine approach against S. pneumoniae, including progression to early-phase clinical trials (11–13). An alternative to maintaining protein antigens as part of the whole S. pneumoniae bacterium is using a bacterial lysate as a vaccine, which could result in a more stable preparation that is better suited to vaccine delivery than a whole bacterium. However, the antigenicity of whole-cell lysates may be weak and require enhancement (14). One method of enhancing immunogenicity is altering the preparation of the lysate to ensure increased representation of immunoprotective proteins. This can be partially achieved using anion-exchange chromatography with a pH 8.0 buffer to preferentially capture several well-known S. pneumoniae antigens, all of which have a pI of 7.5 or lower, including PiuA, PiaA, PsaA, RrgA, RrgB, ClpP, PspA, and Ply. In addition, growth under stress conditions, such as high temperatures, to induce heat shock proteins (Hsps) could increase antigenicity (15), as Hsps facilitate the cross-presentation of peptides (16, 17) and act as natural adjuvants by stimulating macrophages and dendritic cells to cause cytokine secretion (18–20). As a result, Hsps have been studied as vaccines that protect against cancer as well as microbial pathogens (21), with a number of bacterial Hsps showing promise as vaccine candidates (22–24), including in models of lethal lung infection (25–27). For example, mice intranasally immunized with the S. pneumoniae Hsp DnaJ (Hsp40) or Hsp caseinolytic protease P (ClpP) were protected from S. pneumoniae infection, including against systemic challenge with a panel of heterologous strains (28). Hence, Hsps are potential vaccine antigens with advantageous immunomodulatory properties that could be used as a component of a broadly protective S. pneumoniae vaccine.

Here we present data on a multiple-antigen approach to a novel S. pneumoniae vaccine based on bacterial lysates that combines the advantages of a whole-cell approach with the potential additional benefit of an increased Hsp and surface antigen content in the vaccine preparation.

## RESULTS

### Formulation of an S. pneumoniae MAV.

**FIG 1:**
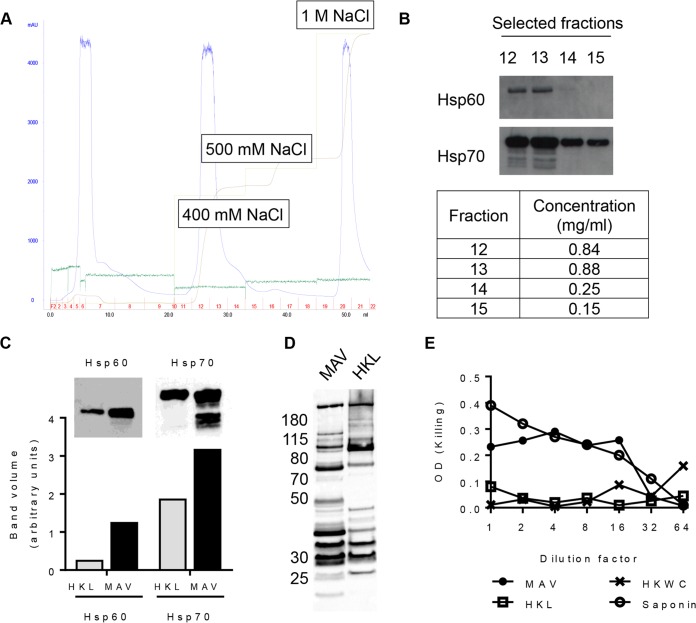
Formulation of a multiple-antigen S. pneumoniae TIGR4-derived vaccine preparation (MAV). (A) Ion-exchange (IEX) chromatogram showing the purification of the MAV. Light green line, NaCl elution concentration; brown line, the resulting conductivity in the system; blue line, UV trace showing the concentrations of the eluted proteins (milli-absorbance units); dark green line, pressure in the system. The fractions collected are numbered in red; the total volume (in milliliters) is recorded on the *x* axis. (B) Detection of Hsp60 and Hsp70 by Western blotting in selected ion-exchange chromatography fractions; the BCA assay protein concentrations for these fractions are shown in the table. (C) A comparison of the heat shock protein content (Hsp60 and Hsp70) measured by immunoblotting of heat-killed lysate (HKL) and MAV. The bar chart shows the pixel intensity quantification (ImageQuant TL; GE Lifesciences) for Hsp60 and Hsp70 bands. (D) Immunoblots of 5 μg of total protein of either MAV or HKL probed with pooled human IgG at 1:20,000 (Pentaglobin; Paviour Pharmaceuticals, New Delhi, India). (E) Comparison of the hemolytic activity against horse red blood cells in serial 2-fold dilutions of MAV from neat to 1:64 and HKL and HKWC preparations with a saponin positive control.

A multiple-antigen Hsp-enriched preparation based on a whole-cell preparation was formulated using the ImmunoBiology Ltd. platform technology as previously described (29). Heat shock was used to enrich for Hsps, and anion-exchange chromatography was used to enrich for negatively charged S. pneumoniae antigens (e.g., PspA and Ply) (Fig. 1A). Immunoblots determined which elution fractions contained the highest concentration of the Hsp60 and Hsp70 proteins and demonstrated a marked increase in the expression of both the Hsp60 and Hsp70 content in the multiple-antigen vaccine (MAV) compared to the bacterial heat-killed lysate (HKL) (Fig. 1B and C). A pooled human IgG preparation known to recognize multiple S. pneumoniae protein antigens (9) was used to probe MAV and HKL to determine whether there were differences in their non-Hsp contents. This demonstrated variations in the number, intensity, and molecular weights of bands identified after incubation in sera from MAV- or HKL-vaccinated animals (Fig. 1D). Ply activity in the MAV, the HKL, and a heat-killed whole-cell (HKWC) preparation formulated with the wild-type TIGR4 strain was assessed using a hemolysis assay. HKWC and HKL did not cause the lysis of red blood cells, probably due to the degradation of Ply during the heat-killing step, whereas MAV caused red cell lysis, suggesting that the MAV preparation still contained active Ply (Fig. 1E).

### Proteomic analysis of MAV preparations.

Tandem mass spectrometry (MS/MS) sequencing was used to identify proteins in the MAV and HKL preparations, with tandem mass tag (TMT) labeling being used to assess relative protein quantities. A total of 627 proteins were identified and compared between the MAV and HKL preparations. Of these, 57 were increased >2-fold in MAV compared to HKL (see Table S1 in the supplemental material), including several Hsps and important known surface antigens, such as PavB and several lipoproteins, including PsaA, PiaA, and the Th17 antigens SP_0148 and SP_2108 (30) (Table 1). Conversely, 152 proteins were decreased by more than 2-fold by the vaccine preparation process, including multiple proteins required for basic metabolic functions (e.g., ribosomal proteins) and capsule synthesis, but also the virulence factors and protective antigens Ply, PspA, and PspC (Table S2). Previously, we have published data obtained using deltaDOT capillary gel electrophoresis (CGE) demonstrating a consistent protein content between different batches of MAV preparations (29).

**TABLE 1:** Selected proteins that TMT-MS/MS analysis shows have increased expression in the MAV TIGR4.1 preparation compared to the TIGR4 HKL preparation^*a*^

SP no.	Protein	Fold change	Reference(s)
Heat shock proteins			
SP_0338	Putative ATP-dependent Clp protease, ATP binding	2.67	51
SP_0517	Chaperone protein (DnaK)	4.33	62, 63
SP_0516	Protein GrpE (GrpE)	3.03	64, 65
SP_0519	Chaperone protein (DnaJ)	2.37	66, 67
SP_1906	60-kDa chaperonin (GroL)	2.95	68
SP_1907	10-kDa chaperonin (GroS)	3.56	69
Known immunogens			
SP_0082	Cell wall surface anchor family protein (PavB)	5.33	70
SP_0148	ABC transporter, substrate-binding protein	3.10	71, 72
SP_0149	ABC transporter substrate-binding protein (MetQ)	2.52	71, 73
SP_0629	l,d-carboxypeptidase (DacB)	3.94	74
SP_0845	Nucleoside ABC transporter protein	2.37	75
SP_1032	Iron compound ABC transporter (PiaA)	2.22	36, 76
SP_1650	Manganese ABC transporter lipoprotein (PsaA)	4.03	77, 78
SP_2093	Putative uncharacterized membrane protein	2.78	79
SP_2108	Maltose/maltodextrin-binding protein (MalX)	3.19	72

a Only proteins with an increased fold change of 2 and above are shown.

### Vaccination with MAV induces functional antibodies.

**FIG 2:**
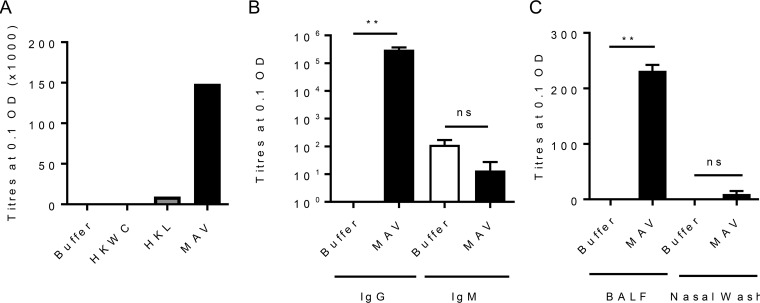
MAV is immunogenic in a mouse model of subcutaneous vaccination. CD1 mice were vaccinated subcutaneously with 75 μg on day 0 and day 21 and culled at 28 days to obtain serum. (A) Results of a whole-cell IgG ELISA against S. pneumoniae TIGR4 for pooled sera harvested from tail vein bleeds (10 μl per mouse, *n* = 6). (B and C) Results of whole-cell IgG and IgM ELISAs against S. pneumoniae TIGR4 for pooled sera from MAV-vaccinated mice (*n* = 5) (B) and against S. pneumoniae TIGR4 for pooled BALF and nasal wash specimens from MAV-vaccinated mice (C). Data are presented as the mean and 95% confidence interval. *P* values were calculated using the Mann-Whitney *t* test. **, *P* < 0.01; ns, not significant.

**FIG 3:**
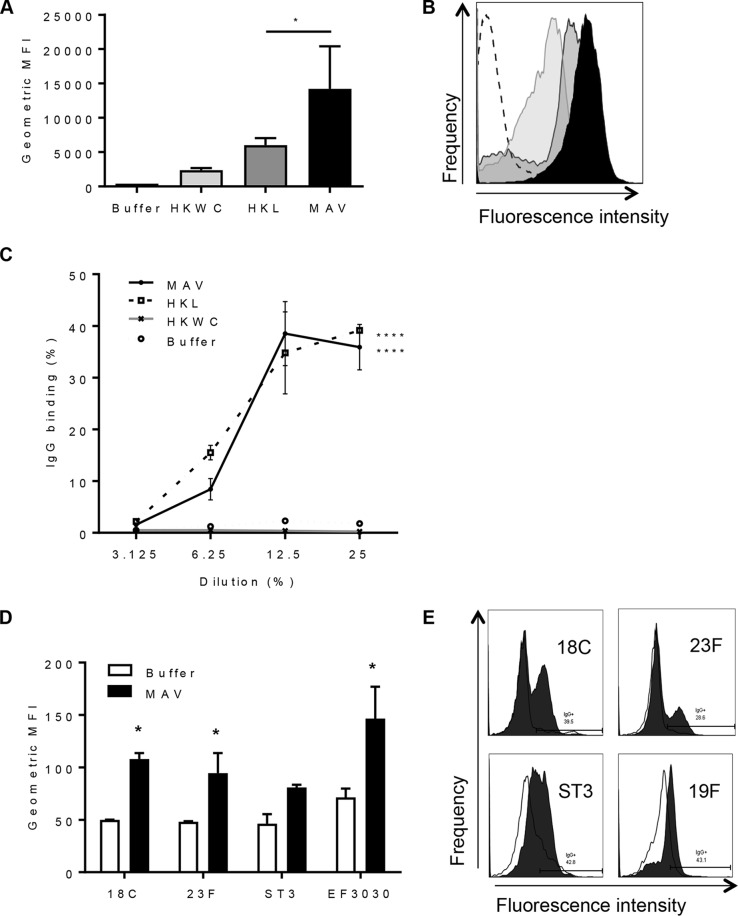
Binding of immune mouse sera to the surface of S. pneumoniae strains. (A) The results of IgG surface binding assays of S. pneumoniae TIGR4 incubated in sera from vaccinated mice are shown as the geometric mean fluorescence index (MFI). Error bars represent the SD from technical replicates. Significance is calculated with the Holm-Sidak test. *, *P* < 0.05. (B) Representative flow cytometry histograms showing IgG-positive S. pneumoniae TIGR4 populations. White histogram, buffer negative-control serum; black histogram, serum from MAV-vaccinated mice; dark gray histogram, serum from HKL-vaccinated mice; light gray histogram, serum from HKWC-vaccinated mice. (C) IgG binding to TIGR4 in immune serum diluted to 25, 12.5, 6.25, and 3.125%. Data points are means from technical replicates; error bars represent standard deviations. Significance values between each dilution curve were calculated by using a two-way ANOVA and comparison to the buffer negative control. ****, *P* < 0.001. (D) Mean fluorescent IgG surface binding to S. pneumoniae serotype 18C, 23F, ST3, and 19F strains incubated in sera from MAV- or buffer-vaccinated mice. Error bars represent standard deviations from technical replicates. Significance was calculated with the Holm-Sidak test. *, *P* < 0.05. (E) Representative histograms showing a shift in populations positive for IgG against different strains of S. pneumoniae. White histogram, IgG binding in buffer-vaccinated mouse serum; shaded histogram, binding in MAV-vaccinated mouse serum.

To assess the immunogenicity of MAV, mice were immunized by subcutaneous injection with either MAV, HKL, HKWC, or the negative-control buffer using a two-dose schedule 21 days apart. Whole-cell enzyme-linked immunosorbent assays (ELISAs) demonstrated that pooled serum obtained 1 week after the second vaccinaton with MAV contained antibody responses to the S. pneumoniae TIGR4 strain markedly higher than those detected in serum from HKL-vaccinated mice. No statistically significant anti-TIGR4 response was identified in sera from mice vaccinated with HKWC (Fig. 2A). The serum antibody response to MAV was dominated by IgG, with no significant IgM response compared to that in buffer-vaccinated mice (Fig. 2B). Significantly increased levels of anti-TIGR4 IgG were also detected in the bronchoalveolar lavage fluid (BALF) of mice immunized with MAV but not in nasal washes (Fig. 2C). Whether the serum IgG induced by the MAV, HKL, and HKWC preparations can recognize and bind to the surface of live S. pneumoniae was assessed using a flow cytometry assay that correlates with protection (31). Compatible with the ELISA data, there were higher levels of IgG binding when S. pneumoniae TIGR4 was incubated in serum from mice vaccinated with MAV than after incubation in serum from mice vaccinated with HKL or HKWC. Incubation in serum from HKWC-vaccinated mice also resulted in less IgG binding to S. pneumoniae than incubation in sera from HKL-vaccinated mice (Fig. 3A to C). To investigate IgG binding to heterologous strains, the IgG binding assays were repeated using S. pneumoniae serotype 18C, 23F, 3, and 19F (EF3030) strains. Sera from MAV-vaccinated mice significantly increased serum IgG binding to the serotype 18C, 23F, 3, and 19F (EF3030) strains compared to that in a buffer-vaccinated control serum (Fig. 3D and E).

### Sera from MAV-vaccinated mice bind to multiple protein antigens.

**FIG 4:**
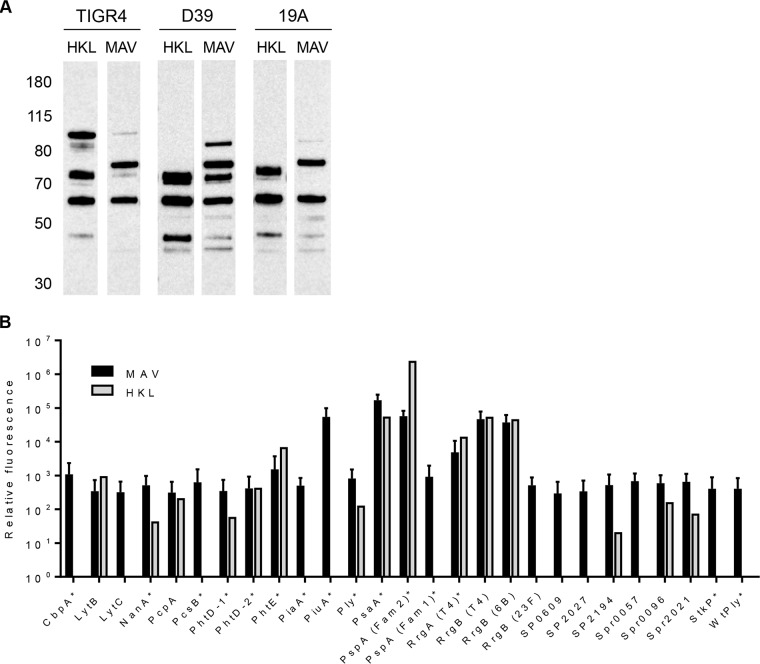
Identification of protein antigens recognized by sera from vaccinated mice. (A) Immunoblots of S. pneumoniae TIGR4, D39, or 19A strain whole-cell lysates probed with serum diluted 1:1,000 from mice vaccinated with either HKL or MAV. The numbers on the left are molecular masses (in kilodaltons). (B) Identification of protein antigens recognized by sera from vaccinated mice using the MSD assay. Values are normalized to those for the negative control consisting of buffer-vaccinated mouse serum. Mean values are shown, with error bars representing standard deviations for sera from mice vaccinated with MAV (*n* = 3, black columns) and a mouse vaccinated with HKL (*n* = 1, gray columns).

Immunoblots against lysates from the S. pneumoniae TIGR4, D39, and 19A strains demonstrated that antibodies in sera from MAV- and HKL-vaccinated mice recognized a number of proteins which were largely conserved between the three strains. The antigens recognized after probing with sera from MAV- and HKL-vaccinated mice overlapped, although a band at approximately 75 kDa (potentially consisting of multiple proteins) was recognized by serum from MAV-vaccinated mice but was not recognized by serum from HKL-vaccinated mice (Fig. 4A). A Meso Scale Discovery (MSD) multiplex assay that measures the levels of IgG to a panel of known S. pneumoniae surface and immunogenic proteins was used to identify some of the protein antigens recognized by sera from vaccinated mice (32). IgG in serum from a MAV-vaccinated mouse recognized all the antigens included in the MSD assay panel of proteins, including PspC (CbpA), PspA, PsaA, PiaA, PiuA, and the pilus proteins RrgA and RrgB, all of which have previously been shown to be protective vaccine candidates in mice (33–36) (Fig. 4B). In contrast, IgG in sera from HKL-vaccinated mice recognized fewer proteins, with no responses to PspC, LytC, PcsB, PiaA, PiuA, family 1 PspA, SP_0609, SP_2027, Spr0057 (StrH), and StkP.

### Comparison of MAV preparations made with and without Hsp induction.

**FIG 5:**
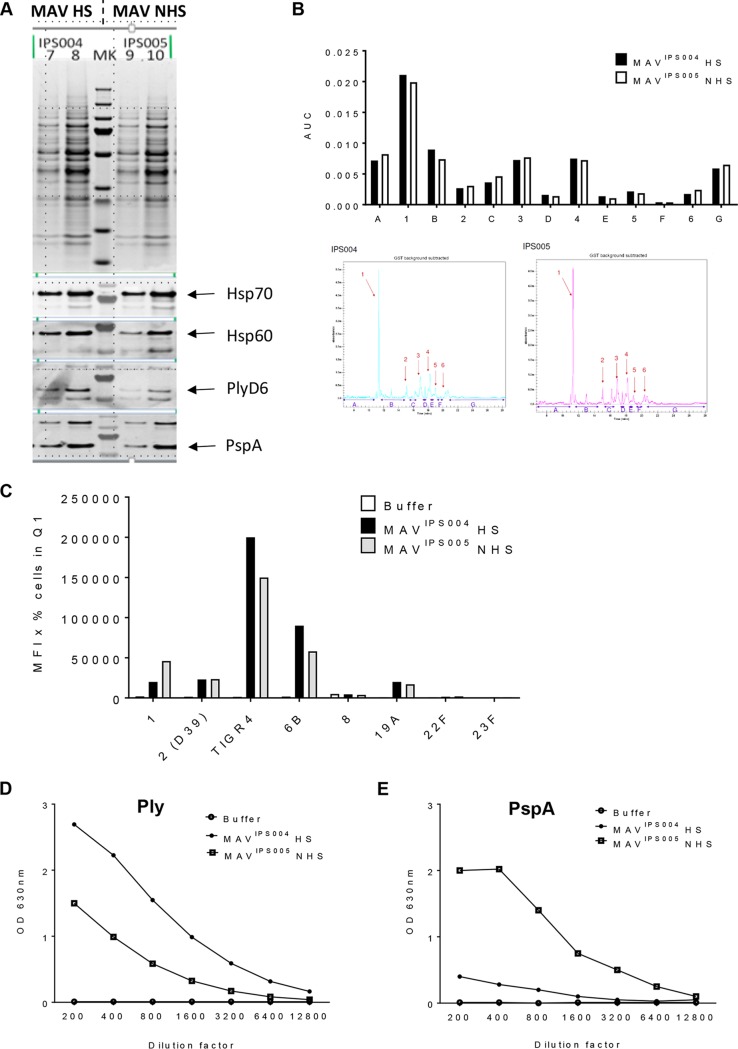
Comparison of heat-shocked MAV versus non-heat-shocked MAV. (A) MAV preparations were made with (MAV^IPS004^) and without (MAV^IPS005^) the Hsp induction step. Protein bands were compared using a Coomassie gel (top). Three micrograms (lanes 7 and 9) and 5 μg (lanes 8 and 10) of each MAV was loaded; immunoblots of the MAV preparations were also probed for the presence of the key S. pneumoniae protein antigens (PlyD6, PspA) and Hsps (Hsp70, Hsp60). (B) Capillary gel electrophoresis (CGE) analysis was conducted to determine the protein constituents of each preparation. Each peak is denoted by a number, and interpeak regions are marked by a letter. Quantification of peaks is shown in the bar chart on top. CGE traces are shown below. (C) Both MAV^IPS004^ and MAV^IPS005^ were used to generate antisera using vaccination experiments in mice. Sera recovered from mice vaccinated with either preparation were analyzed using flow cytometry assays of IgG binding to live S. pneumoniae bacteria (serotypes 1, 2 [D39], 4 [TIGR4], 6B, 8, 19A, 22F, and 23F), and results are represented as the mean fluorescence intensity (MFI) in the appropriate gate. Q1, quadrant 1. (D and E) ELISAs detecting anti-Ply (D) and anti-PspA (E) responses were conducted in duplicate. Sera from the experiments described above were diluted, as shown on the *x* axis, and the OD_450_ was measured for each MAV and a buffer control. Abbreviations: MAV, multiantigen vaccine; HS, heat shocked; NHS, non-heat shocked; MK, molecular weight marker; AUC, area under the curve.

To assess the role of Hsp induction for the immunogenicity of the MAV preparations, MAV preparations were made with (MAV batch IPS004 [MAV^IPS004^]) and without (MAV^IPS005^) the Hsp induction step. Comparing the two MAV preparations using immunoblots showed no clear differences in expression of the Hsps Hsp70 and Hsp60 or in the expression of the immunogenic proteins PspA and Ply (Fig. 5A). The capillary gel electrophoresis profiles of both preparations suggested only minor overall differences in their protein constituents (Fig. 5B). Both preparations were used to generate antisera using vaccination experiments in mice conducted at Churchill Applied Biotechnology Ltd. Sera recovered from mice vaccinated with MAV^IPS004^ and MAV^IPS005^ showed no major statistically significant differences in flow cytometry assays of IgG binding to live S. pneumoniae bacteria or whole-cell ELISA titers against the TIGR4 S. pneumoniae strain (Fig. 5C). Specific protein antigen ELISAs demonstrated increased antibody titers to Ply in serum from mice vaccinated with heat-shocked MAV^IPS004^ compared to those in serum from mice vaccinated with MAV^IPS005^ (Fig. 5D) and reduced titers to PspA (Fig. 5E). When measured using the MSD multiplex assay (32), there were no differences between the two vaccine preparations in responses to other antigens (PiuA, PsaA, RrgA, and RrgB; data not shown). These data indicate that the heat shock step in MAV preparation had limited effects on the heat shock protein content or overall antigenicity.

### Protective efficacy of vaccination of mice with MAV TIGR4 preparations.

**FIG 6:**
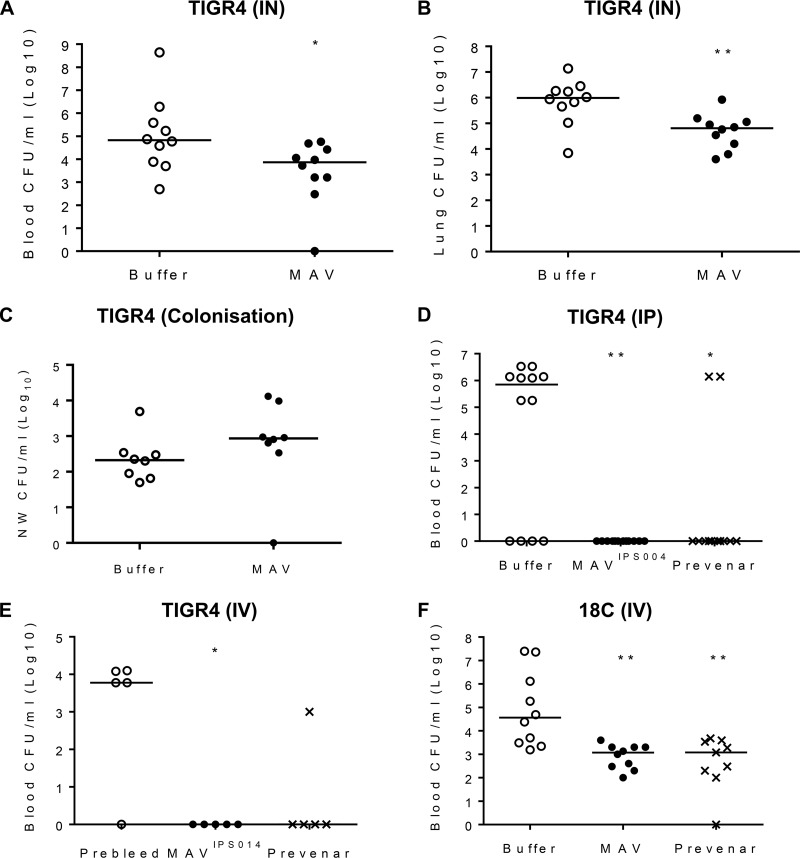
Vaccination with MAV preparations protects mice against S. pneumoniae challenge. (A and B) Number of CFU in the lung (A) and blood (B) 24 h after challenge by intranasal (IN) inoculation with 1 × 10^7^ CFU of the S. pneumoniae TIGR4 strain in mice vaccinated twice subcutaneously with 75 μg of MAV or a negative-control buffer (*n* = 10 per group). (C) Number of CFU in nasal wash (NW) specimens 2 weeks after nasopharyngeal colonization with 5 × 10^6^ CFU of S. pneumoniae TIGR4 of mice vaccinated twice subcutaneously with 75 μg of MAV or a negative control buffer (*n* = 8 per group). (D) Number of CFU in blood 6 h after challenge by intraperitoneal (IP) inoculation of 1 × 10^4^ CFU of the S. pneumoniae TIGR4 strain into mice passively vaccinated with 200 μl of sera from rabbits vaccinated three times with 375 μg of MAV^IPS004^ or twice with 0.2 ml of the Prevenar vaccine or a negative-control buffer (*n* = 12 per group). (E and F) Number of CFU in the blood of mice 4 h after challenge by intravenous (IV) inoculation with 5 × 10^5^ CFU of the S. pneumoniae TIGR4 (E) or ATCC BAA-1662 (18C) (F) strain that was incubated preinoculation in sera obtained from rabbits vaccinated with MAV^IPS014^, the Prevenar vaccine, or a negative-control buffer (*n* = 5 to 10). For all panels, each symbol represents data from a single mouse, and horizontal bars represent median values. Statistical significances were calculated using a Mann-Whitney *t* test (A to D) or Dunnett’s multiple-comparison test (E and F). Significance abbreviations: *, *P* < 0.05; **, *P* < 0.01.

**FIG 7:**
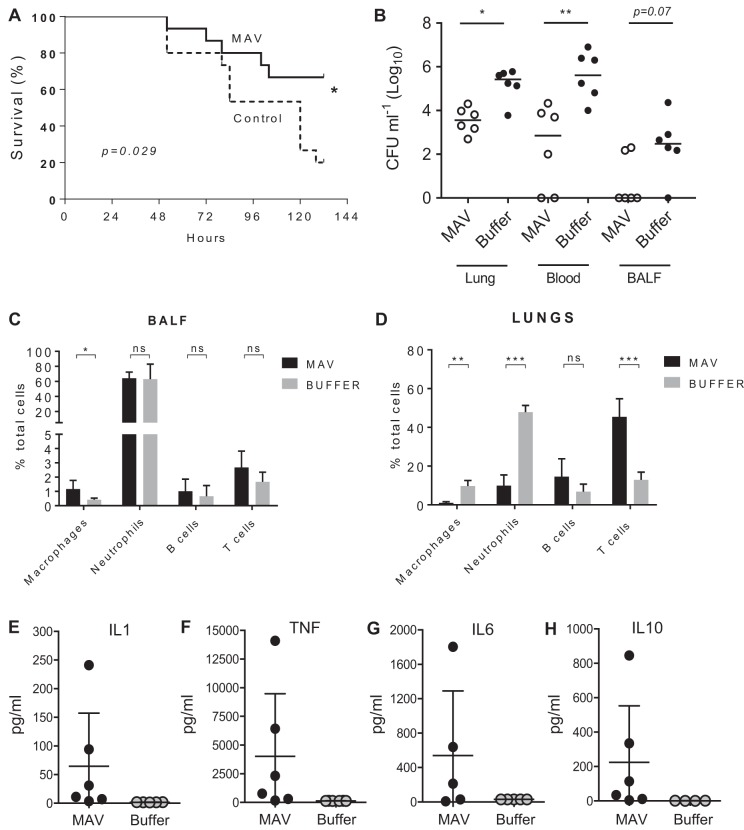
Vaccination with MAV preparations increases the survival of mice and alters the inflammatory response after S. pneumoniae TIGR4 pneumonia challenge. (A) Percent survival of mice over 6 days after challenge by intranasal inoculation with 1 × 10^7^ CFU of the S. pneumoniae TIGR4 strain of mice that had been vaccinated three times (days 1, 10, and 22) intraperitoneally with 75 μg of MAV^IPS014^ or a negative-control buffer (*n* = 15 per group). Significance was calculated using the log-rank (Mantel-Cox) test. (B to D) Numbers of CFU in target organs (B) and inflammatory cell populations in BALF (C) and lung (D) 24 h after challenge with 1 × 10^7^ CFU of the S. pneumoniae TIGR4 strain in MAV^IPS014^-vaccinated and control mice. Inflammatory cell data are shown as a percentage of the total cells recovered from the lungs of MAV- and buffer-vaccinated mice; CFU data show the number of CFU in lung, blood, or BALF recovered 24 h after challenge, with each symbol representing data from a single mouse and horizontal bars representing median values. (E to H) Lung homogenate cytokine levels 24 h after challenge with 1 × 10^7^ CFU of the S. pneumoniae TIGR4 strain in MAV^IPS014^-vaccinated and control mice. For panels B to H, statistical significances were calculated using a Mann-Whitney *t* test. Significance abbreviations: *, *P* < 0.05; **, *P* < 0.01; ***, *P* < 0.001; ns, not significant.

Mouse models were used to determine if vaccination with Hsp-induced MAV resulted in protective immunity against S. pneumoniae. At 24 h after challenge using the pneumonia model, mice vaccinated with MAV had at least 1 log_10_ fewer numbers of bacterial CFU in both the blood and lungs compared to the buffer-vaccinated controls (Fig. 6A and B). In contrast, MAV vaccination did not reduce the density of the number of bacterial CFU in nasal washes obtained 2 weeks after inoculation in a model of S. pneumoniae nasopharyngeal colonization with TIGR4 (Fig. 6C). In order to eliminate potential adverse effects caused by an active Ply, new MAV preparations, termed MAV^IPS004^ and MAV^IPS014^, denoting different batches, were prepared using a mutated TIGR4 strain expressing a detoxified Ply (37). Both MAV^IPS004^ and MAV^IPS014^ contained similar levels of detoxified Ply, as measured by ELISA, that stimulated an antibody response that recognized native Ply (29) and the absence of hemolysis in a red blood cell assay. Sera obtained from rabbits vaccinated subcutaneously on days 0, 21, and 35 with 375 μg of MAV^IPS004^, the S. pneumoniae vaccine Prevenar as a positive control, or buffer were used for passive immunization of mice, followed by intraperitoneal challenge with 1 × 10^4^ CFU of TIGR4 S. pneumoniae after 6 h. When culled at 24 h postchallenge, colonies were recovered from the blood of over 65% of the mice given sera from buffer-vaccinated rabbits, while there no colonies were detected in mice given sera from MAV^IPS004^-vaccinated rabbits (Fig. 6D). Two mice (16%) developed septicemia after passive administration of sera from rabbits given the Prevenar vaccine. In an alternative sepsis model, mice were challenged by intravenous inoculation with 5 × 10^5^ CFU of S. pneumoniae TIGR4 or ATCC BAA-1662 (serotype 18C S. pneumoniae) strains after preincubation of the bacteria for 1 h in 100% serum obtained from rabbit immunized with MAV^IPS014^, the Prevenar vaccine, or buffer controls. Mice were culled at 4 h to assess the rate of bacterial clearance from the blood by quantifying the number of CFU. Those challenged with the TIGR4 strain were almost completely protected against infection if the bacteria were incubated in sera from either MAV^IPS014^- or Prevenar-vaccinated rabbits prechallenge (Fig. 6E). Preincubation of the 18C strain prior to intravenous challenge in sera from either MAV^IPS014^- or Prevenar-vaccinated rabbits prechallenge reduced the number of bacterial CFU recovered from the blood by over 1 log_10_ compared to the number obtained with preincubation in sera from buffer-vaccinated rabbits (Fig. 6F). To support these data, vaccination with MAV^IPS014^ was compared to that with a buffer control in a protection study, which demonstrated that mice given a three-dose vaccination schedule with MAV^IPS014^ were protected against the development of fatal infection after pneumonia challenge with TIGR4 (Fig. 7A). The effects of vaccination on the inflammatory response to pneumonia challenge was assessed using flow cytometry of lung and BALF cell populations 24 h after intranasal infection with TIGR4. Despite clear reductions in the numbers of CFU in the lungs and blood of vaccinated mice, there were no differences in the proportions of neutrophils in BALF between MAV- and buffer-vaccinated mice, indicating that MAV vaccination resulted in an increased BALF neutrophil response for the level of bacterial infection (Fig. 7B and C). In addition, in lung homogenates, MAV-vaccinated mice had reduced proportions of neutrophils and macrophage lineage cells and a corresponding increase in T cells (Fig. 7D). The increase in the T cell proportion within lung homogenates in vaccinated mice did not alter the CD4/CD8 proportions compared to the data for control mice (data not shown). Lung homogenate cytokine levels were variable between mice but showed increased interleukin-1 (IL-1), IL-6, tumor necrosis factor alpha (TNF-α), and IL-10 responses in vaccinated mice compared to the controls, again suggesting that vaccinated mice were able to mount a more sustained inflammatory response than the controls (Fig. 7E to H). BALF and blood cytokine levels were, in general, too low and variable for consistent patterns to be identified.

Overall, these results indicate that the MAV is able to protect against S. pneumoniae infection with the homologous strain or a heterologous strain at a level of protection similar to that provided by the Prevenar vaccine and is associated with significant changes in the inflammatory response to pneumonic infection.

## DISCUSSION

Although the existing childhood conjugated capsular polysaccharide S. pneumoniae vaccines are highly effective, both the lack of protection against nonvaccine serotypes and convincing evidence of serotype replacement in vaccinated populations, as well as the high cost of these vaccines, have stimulated continued interest in alternative vaccine approaches (38, 39). Vaccines based on protein antigens could overcome these disadvantages, allowing production of relatively inexpensive vaccines that target cross-protective antigens (40–43). Presentation of a large number of protein antigens derived from a whole-cell approach has the additional potential advantages of inducing immune responses to multiple antigens, thereby potentially avoiding the development of vaccine escape mutants and inducing stronger cross-protective responses (44, 45).

The data presented here show that a novel S. pneumoniae multiple-protein-antigen vaccine approach induces antibody that recognizes homologous and heterologous strains and protects against invasive pneumonia and sepsis. The MAV approach uses a whole-cell lysate that has been manipulated to increase the expression of Hsps, and the anion-exchange column and running buffer are optimized for the capture of known S. pneumoniae antigens as well as Hsps. Proteomic analysis confirmed that the MAV approach alters the relative levels of S. pneumoniae proteins within the preparation compared to those in a simple bacterial lysate, with altered expression of a total of 209 proteins of the 627 proteins analyzed. The S. pneumoniae TIGR4 strain actually contains approximately 2,000 genes (46), so the number of proteins with altered content in the MAV may, in fact, be substantially larger. Both immunoblotting and TMT-MS confirmed that the MAV had an increased Hsp content compared to that of a simple lysate. In addition, there was increased expression of multiple surface proteins, including known protective antigens (mainly lipoproteins), although there were also reduced amounts of other protein antigens that are known to induce protective immunity in mice.

Importantly, whole-cell ELISAs, the MSD assay for IgG responses to individual antigens, and a flow cytometry assay of IgG binding to S. pneumoniae all demonstrated enhanced antibody responses in mice vaccinated with the MAV compared to the HKL, demonstrating the benefit of the MAV approach in making a potentially more effective vaccine. Unlike HKL, MAV induced antibody responses to all the protein antigens tested using the MSD system, suggesting that the MAV approach may induce antibody responses to a very high proportion of the proteins in the preparation. Furthermore, although iTRAQ demonstrated a reduced content in the MAV preparation compared to that in HKL for the important antigens PspC and Ply, vaccination with MAV still induced stronger IgG responses to these antigens, when tested using the MSD assay, than vaccination with HKL. These data again suggest that the MAV approach enhances immunogenicity. The improved serological responses to MAV compared to HKL might be predicted to be due to the increased Hsp content leading to the formation of Hsp-peptide complexes and thereby increasing the antigen-presenting ability (27, 29). However, direct comparison of MAV preparations with or without a heat shock step as vaccines in mice did not show any major differences in protein content (including the Hsp60 and Hsp70 content) or in immunogenicity, apart from a reduction in the antibody responses to PspA. These data suggest that the anion-exchange chromatography step alone enhances the immunogenicity of the MAV preparations and seems to increase the heat shock protein content independently of the heat shock step. In addition, the lower temperature used for making MAV preparations compared to that required for making HKL preparations could have allowed some proteins to retain stronger immunogenicity.

Data from a pneumonia model and two separate mouse models of sepsis demonstrated that active or passive vaccination with MAV improved protection against S. pneumoniae. Time course experiments demonstrated that vaccination with the MAV preparation delayed and protected against lethal infection. The total T cell populations in the lung were also increased in MAV-vaccinated mice. This effect in T cell proportions probably reflects the accumulation of antigen-specific cells during pneumonia in infected tissue, but further experiments would be needed to confirm this. Overall, the data demonstrate that vaccination with MAV had significant effects on the inflammatory response during S. pneumoniae pneumonia that are likely to contribute to protective efficacy, with improved neutrophil recruitment into the alveolar space and increased numbers of T cells within the lung parenchyma.

MAV also induced IgG that bound to heterologous strains in the flow cytometry assay, an assay which correlates with improved phagocytosis *in vitro* and protection in mouse models (31, 47, 48). Indeed, in a mouse model of sepsis, passive vaccination with sera from MAV-vaccinated rabbits gave a level of protection against both the homologous TIGR4 and heterologous 19C strains similar to that achieved with passive vaccination with sera from Prevenar-vaccinated rabbits. Whether vaccination with MAV can induce stronger protection than vaccination with HKL or HKWC in the mouse models was not tested; due to the relatively low sensitivity of the mouse models, this is likely to require inappropriately large numbers of mice per test group. A lack of sensitivity is also why vaccination followed by disease challenge experiments would be unlikely to show any significant differences between MAV preparations with and without heat shock steps and why these experiments were not performed. The level of protection varied between models, with complete prevention of septicemia being seen in some models, e.g., intravenous TIGR4 infection, but reductions in the number of bacterial CFU only in the blood being seen in others (e.g., the serotype 18C sepsis model). The latter is likely to slow the progression of disease but not prevent fatal infection. Vaccination with MAV failed to protect against colonization, but this is not that surprising, given the lack of detectable antibody in nasal washes and previous data showing that antiprotein antibody is often ineffective at reducing the number of nasopharyngeal S. pneumoniae CFU (in some cases, even when Th17-mediated immunity has also been induced) (9, 10, 49). Prevention of nasopharyngeal colonization will probably require vaccines that induce strong cell-mediated immune mechanisms, which may require vaccination in combination with specific adjuvants (24, 46, 50). This would be an important area for further investigation. Future experiments should also assess whether MAV vaccination modulates pulmonary inflammatory responses to S. pneumoniae pneumonia challenge.

The MAV approach described here can induce the cross-protective immunogenicity of protein antigens which stimulate antibody and perhaps Th17 cell responses (30, 51) without requiring the identification of specific protective antigens or the production of recombinant proteins for inclusion in subunit vaccines. MAV requires limited downstream processing and rapidly produces a high yield of vaccine product, considerably reducing vaccine costs and making the vaccine more likely to be affordable in low- and middle-income countries. The MAV approach therefore offers a promising opportunity for a novel next-generation S. pneumoniae vaccine and has recently completed a phase I trial in 36 subjects (ClinicalTrials.gov registry number NCT0257635 [52]).

## MATERIALS AND METHODS

### Bacterial strains and growth conditions.

S. pneumoniae was grown in either Todd-Hewitt medium containing 5% yeast extract (THY; Oxoid, UK) or defined Hoeprichs’ medium and on 5% blood Columbia agar (Oxoid) plates containing 5% defibrinated horse blood (TCS Biosciences, UK) at 37°C in 5% CO_2_. Growth in medium was assessed using the optical density at 580 nm (OD_580_) − OD_600_, with bacterial stocks grown to mid-log phase (OD_580_, 0.4 to 0.5) before storage in 10% glycerol at −80°C. Bacterial counts were determined as previously described (53–55) by plating 10-fold serial dilutions of aliquots on 5% blood Columbia agar plates after overnight incubation at 37°C in 5% CO_2_. The TIGR4 strain-derived multiple-antigen vaccine (MAV) was made from S. pneumoniae TIGR4 (American Type Culture Collection [ATCC] strain ATCC BA-334), and MAV batches IPS004, IPS005, and IPS014 were made from strain TIGR4 B7.1 (PlyD6), which expresses an inactivated pneumolysin toxin, made as previously described (29). Additional S. pneumoniae strains used in this study were D39 (serotype 2); 0100093 (serotype 3); 23F, a gift from B. Spratt (Imperial College London); 18C, from ATCC (ATCC BAA-1662); EF3030 (serotype 19F), a gift from D. Briles (University of Alabama); and strain 1777/39 (19A), a gift from J. Paton (University of Adelaide).

### MAV, heat-killed lysate, and heat-killed whole-cell preparation.

MAV were made from S. pneumoniae TIGR4 cultured in Hoeprichs’ medium (made in-house) in 1-liter shake flasks at 37°C to an of OD_600_ of 1.2 before it was heat shocked at 42°C for 30 min. This step was omitted for the non-heat-shocked preparation, MAV^IPS005^. The bacteria were then centrifuged twice with wash buffer (40 mM Tris, 150 mM NaCl, 1 mM MgCl_2_, pH 8.0) and incubated with lysis buffer (40 mM Tris, 20 mM NaCl, 1 mM MgCl_2_, 0.5% [wt/vol] sodium deoxycholate [NaDOC], pH 8.0) for 1 h at 4°C, before homogenization (EmulsiFlex C5 high-pressure homogenizer; Avestin, Germany) and incubation with 0.1% (wt/vol) octaethylene glycol monododecyl ether (C_12_E_8_) for 4 h at 4°C. Sample supernatants were harvested using a 5-ml Capto Q column (GE Healthcare, UK). Protein was eluted and collected as 5-ml fractions at 400 mM and 500 mM concentrations of NaCl. The IPS004 and IPS014 MAV batches were made as described above using the TIGR4 B7.1 strain (PlyD6) with heat shock at 37°C for 30 min and lysis of IPS004 in NaDOC (0.5%) and C_12_E_8_ (0.1%) and of IPS014 in NaDOC (0.5%) and Triton X-100 (1%). For heat-killed lysates (HKL) of TIGR4 and heat-killed whole-cell (HKWC) preparations, TIGR4 was also cultured in Hoeprichs’ medium in 1-liter shake flasks at 37°C to an OD_600_ of 1.2, followed by killing by incubation at 65°C for 45 min, which was confirmed by culture on Columbia agar plates. For the HKL, lysis was performed using NaDOC and high-pressure homogenization as described above. C_12_E_8_ was added to both the HKL and HKWC before filtration.

### Vaccine characterization.

Vaccine samples were analyzed with SDS-PAGE using a 4 to 12% NuPAGE gel (Invitrogen, USA), MES (morpholineethanesulfonic acid; Invitrogen) running buffer, and staining for protein with InstantBlue (Expedeon, UK). For Western blot analysis, gels were subsequently blotted onto polyvinylidene difluoride (PVDF) membranes and probed with the appropriate antibodies diluted in 5% milk–phosphate-buffered saline (PBS): anti-Hsp60 (GroEL; catalog number SPS-875; StressGen, USA) at 1:2,000, antipneumolysin (catalog number ab49568; Abcam, UK) at 1:2,000, anti-Hsp70 (made in-house) at 1:500, and anti-PspA (catalog number sc17483; Santa Cruz, USA) at 1:1,000. Protein concentrations were determined using bicinchoninic acid (BCA) protein assays (Pierce, USA). For hemolysis assays, the vaccine preparations were serially diluted in PBS, an equal volume of 2% defibrinated horse blood was added, and the mixture was incubated at 37°C for 30 min, followed by centrifugation at 1,000 × *g* for 1 min and measurement of the absorbance of the supernatants at 490 nm.

### Capillary gel electrophoresis.

Analysis of the samples by capillary gel electrophoresis (CGE) was conducted by deltaDOT, London BioScience Innovation Centre, using the high-performance capillary electrophoresis (HPCE) platform Peregrine. Peaks were manually selected, and raw data are expressed as the peak area corrected for migration time and then expressed as a percentage of the total corrected peak area or the area under the curve (inclusive of the Triton X-100 peak [peak 1] and interpeak regions [regions A to G]) normalized to an external protein standard. This compensates for day-to-day variation and allows comparison between runs on different sections of the capillary. Peak 1 is the detergent Triton X-100 peak.

### *In vitro* assays.

S. pneumoniae whole-cell ELISAs were performed using bacterial cultures at an OD_580_ of 0.4 to 5, alkaline phosphatase (AP)-conjugated secondary antibodies, and the substrate *para*-nitrophenylphosphate (pNPP; Sigma) as previously described (56, 57). The absorbance was read at 450 nm, and the readings obtained at 630 nm were subtracted (VersaMax). The ELISA titer represents the theoretical sample dilution that would result in an OD_450_ reading minus OD_630_ reading of 0.1. For detection of anti-Ply and anti-PspA antibodies, the appropriate antigen was diluted in carbonate buffer to a final concentration of 1 μg/ml, 100 μl was transferred to each well of a 96-well MaxiSorp ELISA plate, and the plate was incubated overnight at 4°C. The plates were then washed 3 times with ELISA wash buffer (1% [vol/vol] Tween 20–PBS), blocked for 1 h with block buffer (1% [wt/vol] bovine serum albumin [BSA]–PBS) at 37°C, and then washed as described above. Serum samples were diluted to a starting dilution of 1/100 to 1/300. Doubling dilutions of the pooled serum samples were assayed in duplicate. The plates were incubated at 37°C for 1 h and washed, and goat anti-mouse IgG-horseradish peroxidase diluted to 1/20,000 was added before incubation at 37°C for 1 h before washing. Tetramethylbenzidine substrate was added before incubation at room temperature in the dark. The plates were read at OD_450_, and endpoint titers were calculated using the linear part of each titration curve. IgG surface binding was assessed using previously described flow cytometry assays (31, 47, 48) and species-appropriate secondary antibodies: anti-human IgG secondary antibody (1:200) conjugated to phycoerythrin (PE; Sigma-Aldrich) and goat anti-mouse IgG conjugated to fluorescein isothiocyanate (Bio-Rad, USA). Fluorescence-activated cell sorting (FACS) analysis of bacterial cells was performed on a FACSVerse flow cytometer (Becton, Dickinson, USA) and with FACSuite (Becton, Dickinson) and FlowJo (Becton, Dickinson) software. FACS surface binding comparisons of MAV^IPS004^ and MAV^IPS005^ were conducted by ImmBio, as follows: 100 μl of bacterial suspension was placed in each 5-ml FACS tube, and the tubes were incubated overnight at 4°C. Cells were washed with PBS–0.1% Tween 20 (PBS-T). Preadsorbed serum samples were serially diluted (2-fold), starting from 1 in 25, to 1 in 800 in PBS–1% BSA. Cells and sera were incubated together for 2 h at room temperature and then washed with PBS-T. Goat anti-mouse IgG detection antibody in PBS–1% BSA was added, and the mixture was incubated for 2 h. Cells were then fixed with formalin for 30 min at room temperature. Following washing in PBS-T, samples were resuspended in PBS–1% fetal calf serum. The mean fluorescence intensity (MFI) was read by flow cytometry, with 100,000 events being required for each sample. The MFI multiplied by the number of cells in quadrant 1 is shown for each serotype. This represents the degree of antibody binding to each serotype. Multiplexed electroluminescence assays were conducted as previously described (32, 58, 59) using a Meso Scale Discovery (MSD; MD, USA) platform assay (60) with 5 μg/ml of S. pneumoniae proteins and 10 μg/ml of capsular polysaccharide. After incubation of each antigen-coated plate with blocking agent, washing, and incubation with diluted test serum for 45 min at room temperature, the plates were washed and MSD assay sulfo tag-labeled goat anti-mouse IgG secondary antibody was added for reading using an MSD Sector Imager 2400 or 6000 apparatus.

### Quantitative comparison of protein content using TMT and MS.

The tandem mass tags (TMT) labeling procedure followed the manufacturer’s recommendation (Thermo Fisher). In brief, protein lysates from two replicates of HKL and MAV were reduced with tris(2-carboxyethyl) phosphine and alkylated with iodoacetic acid before an overnight acetone precipitation. Protein pellets were digested overnight at 37°C in 200 mM TEAB solution containing 2.5 μg trypsin (Promega), with the resulting peptides being labeled with different isobaric tags (TMTs 126 to 128). Labeled peptides were mixed and injected onto an XBridge C_18_ column (particle size, 5 μm; 4.6 mm [inside diameter] by 25 cm long; Waters) for first-dimension high-pH reversed-phase high-performance liquid chromatography (HPLC) separation under a linear gradient consisting of mobile phase A (10 mM ammonium formate, pH 10.0) and up to 70% mobile phase B (90% acetonitrile in mobile phase A) for 2 h at flow rate of 0.5 ml/min, using a Jasco system consisting of an autosampler, semimicro-HPLC pumps, and a UV detector. The eluted fractions were collected, concatenated into 18 tubes, and vacuum dried.

Nano-liquid chromatography and tandem mass spectrometry (MS/MS) were performed using a U3000 direct nano system coupled with a nano electrospray and LTQ-Orbitrap Discovery mass spectrometer (Thermo). The 12 HPLC fractions containing the mixture of fourplex-labeled peptides were resuspended in 0.1% formic acid, and each was separated on a PepMap C_18_ reversed-phase nano column (particle size, 3 μm; 100 Å; 50-cm length; Thermo) under a column flow rate of 0.3 μl/min using a linear gradient of 95% acetonitrile and 0.1% formic acid at 5 to 25% for 180 min, 25 to 32% for 20 min, and 32 to 90% for 10 min. MS scan and MS/MS fragmentation were carried out in the LTQ-Orbitrap Discovery mass spectrometer, using 2 cycles of top 3 data-dependent acquisition with the dynamic exclusion mode enabled and a total cycle time of approximately 30 ms. The first cycle used collision-induced dissociation (CID) fragmentation-generating spectra for peptide sequencing, and the second used high-energy-CID (HCD)-generating spectra both for peptide sequencing and for relative quantitation via report ions.

Mass spectrum processing, database searching, and quantitation against the UniProt S. pneumoniae FASTA database (release 2014.04.03) were performed using Thermo Proteome Discoverer (version 1.4) software with a built-in Sequest program. Spectra from the 12 fractions were added together as one sample during the search. Initial mass tolerances by MS were set to 10 ppm. Up to two missed tryptic cleavages were considered. Methionine oxidation was set as a dynamic modification, whereas carboxymethylation on cysteine and TMT 6-plex labels on the N-terminal amino acid and the lysine side chain were set as static modifications. Peptides at rank 1 with a high confidence were considered to be unambiguously sequenced. Quantification was based on the relative abundances of the TMT tag as the reporter ions for each peptide in the HCD spectra with all TMT channels present. Ratios were calculated from the relative abundances of each labeled peptide in the sample based on reporter ion intensities, and for every protein identified, each was assigned a series of quantification ratios relative to each group.

### *In vivo* methods.

All *in vivo* experiments using mice were performed according to United Kingdom national guidelines for animal use and care. Experiments performed at UCL were approved by the UCL Biological Services Ethical Committee and the UK Home Office (project license PPL70/6510). Experiments used 6-week-old outbred female CD1 mice obtained from Charles River Laboratories. Mice were vaccinated with 75 μg of protein in 100 μl PBS using either intraperitoneal injection at days 0, 14, and 28 or subcutaneous vaccination on days 0 and 21. Tail bleeds (5 μl per mouse) were collected on day 42, and mice were challenged with S. pneumoniae on day 49. For the pneumonia model, mice under isoflurane (4%; MiniRad) anesthesia were inoculated with 5 × 10^6^ CFU of S. pneumoniae in PBS intranasally. After either 24 or 48 h, the mice were euthanized with pentobarbitone, and blood, serum, bronchoalveolar lavage fluid (BALF), the lungs, and the spleen were collected as previously described (9, 31, 50, 61). Lungs and spleens were macerated through a 0.2-μm-pore-size filter. For the colonization model, mice were anesthetized with aerosolized isoflurane (4%) and inoculated with 5 × 10^6^ CFU of S. pneumoniae suspended in 10 μl of PBS. At designated time points postinfection, the mice were culled and nasal washes were obtained by retrograde washing of the nares with 500 μl PBS via the trachea. To assess survival, mice were vaccinated by intraperitoneal inoculation with 75 μg of the MAV^IPS014^ vaccine together with the adjuvant system (catalog number S6322; Sigma) on days 1, 10, and 22 before intranasal challenge with 1 × 10^7^ CFU of S. pneumoniae TIGR4 on day 50. Disease development was monitored over 6 days, and mice were culled when they exhibited signs of severe disease (61). For the passive transfer model, mice were injected intraperitoneally with 200 μl of serum harvested from rabbits vaccinated by subcutaneous injection of 375 μg MAV on days 0, 21, and 35 or two 200-μl Prevenar13 doses on days 0 and 21 at Envigo, UK. Mice were challenged 6 h later by intraperitoneal inoculation of 1 × 10^4^ CFU of S. pneumoniae and culled at 24 h to obtain blood samples for plating. For the sepsis model, sera from vaccinated mice or rabbits were transferred to mice via intravenous injections into the lateral tail vein. After 4 h, the mice were inoculated intravenously with 5 × 10^5^ CFU of S. pneumoniae and culled 4 h later to collect blood. For the preopsonization clearance model, S. pneumoniae bacteria were opsonized by incubation in 100% rabbit immune serum for 1 h at 37°C, and then 5 × 10^5^ CFU of S. pneumoniae was inoculated intravenously into the mice, which were then culled 4 h later to obtain blood for CFU quantification by plating. To calculate the numbers of target organ CFU, aliquots of blood and lung and spleen tissues were plated at appropriate dilutions on 5% blood Columbia agar plates containing 5 mg/ml gentamicin (Sigma). Additional experiments to raise antisera with different vaccine preparations were performed at a commercial organization, Churchill Applied Biotechnology Ltd., according to institutional guidelines under its UK Home Office project license. For these experiments, six groups of female CD-1 mice (*n* = 10) were immunized subcutaneously with 0.75 μg of MAV on day 0 and day 21. Mice were culled on day 35 and terminally bled, and serum was prepared for the investigation of antibody responses.

### Flow cytometry phenotypic screening of inflammatory cell populations.

The lungs of vaccinated mice were harvested 24 h after infection, and single-cell suspensions were prepared by homogenizing the tissues and filtering them with 100-μm-mesh-size cell strainers. Red blood cells were lysed with red blood cell lysis buffer (catalog number 420301; BioLegend), and washed cells were resuspended at a concentration of 10^6^ cells/ml in blocking buffer (PBS–1% BSA containing anti-Fc receptor antibodies [TruStain FcX; BioLegend]). The cells were seeded in round-bottom 96-well plates (100 μl/well) and incubated for 30 min on ice. Cells were washed and stained with a mixture of antibodies diluted 1:100 for 30 min in ice. The antibodies used were anti-mouse CD19 Brilliant Violet 480 (catalog number 566167; BD Bioscience), anti-mouse CD11c PE-Cy7 (catalog number 117317; BioLegend), anti-mouse Ly-6G peridinin chlorophyll protein-Cy5.5 (catalog number 127615; BioLegend), anti-mouse F4/80 Brilliant Violet 421 (catalog number 123131; BioLegend), anti-mouse CD3 PE (catalog number 100205; BioLegend), and anti-mouse CD4 allophycocyanin (APC) and anti-mouse CD8 APC-Cy7 (catalog number 100713; BioLegend). The cells were washed three times with PBS and stained with a Zombie Green Fixable viability kit (catalog number 423111; BioLegend) (1:500) for 15 min according to the manufacturer’s instructions. After two extra washes with PBS–1% BSA, the cells were fixed with paraformaldehyde (PFA). The samples were analyzed on a FACSVerse flow cytometer (BD Bioscience). The neutrophil/monocyte population and the lymphocyte populations were initially identified using forward and side scatter dimensions, and the immune subpopulations were defined as follows: macrophages, CD11c^+^ F4/80^+^ Ly-6G^−^; neutrophils, Ly-6G^+^ CD11c^−^ F4/80^−^; B cells, CD19^+^ CD3^−^; and T cells, CD3^+^ CD19^−^. T cell populations were further subdivided using CD4 and CD8 markers. Lung homogenate cytokine levels (IL-1, IL-6, IL-10, TNF-α) were determined by using a Luminex magnetic bead array assay (R&D Systems) according to the manufacturer’s protocols.

### Statistical methods.

Statistical analyses were conducted using Prism (version 7) software (GraphPad, USA). Parametric data are presented as means, and error bars represent standard deviations (SD). Comparisons between multiple groups were conducted using analysis of variance (ANOVA) and the Holm-Sidak or Dunnett’s posttest to compare experimental groups. Nonparametric data were analyzed using the Mann-Whitney U test. For the disease development model, data were analyzed using the log-rank (Mantel-Cox) test.

## Supplementary Material

Supplemental file 1
