# Supplementary material for: A Novel, Multiple-Antigen Pneumococcal Vaccine Protects against Lethal Streptococcus pneumoniae Challenge
Source: Infect Immun. 2019 Feb 21;87(3):e00846-18. doi: 10.1128/IAI.00846-18 (PMC6386546; doi:10.1128/IAI.00846-18)
Supplement: Supplemental file 1 [file IAI.00846-18-s0001.pdf]

Supplementary Table 1

All proteins that iTRAQ analyses show have increased expression in the MAV TIGR4.1 compared to the TIGR4 HKL preparation

SP #	Protein	Fold change
SP_0082	Cell wall surface anchor family protein (PavB)	5.33
SP_0148	ABC transporter, substrate-binding protein	3.10
SP_0149	Lipoprotein	2.52
SP_0285	Alcohol dehydrogenase, zinc-containing	2.90
SP_0338	Putative ATP-dependent Clp protease, ATP-binding	2.67
SP_0409	Putative uncharacterized protein	2.96
SP_0447	Ketol-acid reductoisomerase (IlvC)	2.66
SP_0466	Putative sortase	3.42
SP_0516	Protein GrpE (GrpE)	3.03
SP_0517	Chaperone protein (DnaK)	4.33
SP_0629	Putative uncharacterized protein	3.94
SP_0641	Serine protease, subtilase family	3.12
SP_1027	Putative uncharacterized protein	3.12
SP_1190	Tagatose 1,6-diphosphate aldolase (LacD)	4.74
SP_1192	Galactose-6-phosphate isomerase subunit (LacB)	3.13
SP_1415	Glucosamine-6-phosphate deaminase (NagB)	2.62
SP_1572	Non-heme iron-containing ferritin	2.6
SP_1650	Manganese ABC transporter lipoprotein (PsaA)	4.03
SP_1676	Putative N-acetylneuraminate lyase	3.06
SP_1775	Conserved domain protein	2.79
SP_1776	Thioredoxin (Trx)	2.96
SP_1906	60 kDa chaperonin (GroL)	2.95

Supplementary Table 1 continued		
**SP #**	**Protein**	**Fold change**
SP_1907	10 kDa chaperonin (GroS)	3.56
SP_2055	Alcohol dehydrogenase, zinc-containing	3.46
SP_2056	N-acetylglucosamine-6-phosphate deacetylase	3.24
SP_2093	Putative uncharacterized protein	2.78
SP_2108	Maltose/maltodextrin-binding protein (MalX)	3.19
SP_2239	Serine protease	3.29
SP_1069	Putative uncharacterized protein	2.51
SP_2126	Dihydroxy-acid dehydratase (IlvD)	2.48
SP_0519	Chaperone protein (DnaJ)	2.37
SP_0845	Lipoprotein	2.37
SP_1500	Amino acid ABC transporter, (AatB)	2.37
SP_1527	Oligopeptide-binding protein (AliB)	2.33
SP_0771	Peptidyl-prolyl cis-trans isomerase, cyclophilin-type	2.31
SP_1683	Sugar ABC transporter, sugar-binding protein	2.3
SP_0899	Putative uncharacterized protein	2.26
SP_2070	Glucose-6-phosphate isomerase (Pgi)	2.24
SP_1032	Iron-compound ABC transporter	2.22
SP_0736	Mannose-6-phosphate isomerase (ManA)	2.22
SP_0520	Putative uncharacterized protein	2.21
SP_0605	Fructose-bisphosphate aldolase (Fba)	2.20
SP_2197	Putative ABC transporter, substrate-binding protein	2.17
SP_0236	DNA-directed RNA polymerase subunit alpha (RpoA)	2.17
SP_0372	Cell cycle protein GpsB (GpsB)	2.16
SP_0673	Putative uncharacterized protein	2.15
SP_1465	Putative uncharacterized protein	2.12

Supplementary Table 1 continued		
**SP #**	**Protein**	**Fold change**
SP_1996	Universal stress protein	2.11
SP_0374	Putative uncharacterized protein	2.11
SP_1177	Phosphocarrier protein HPr (PtsH)	2.10
SP_1541	30S ribosomal protein S6 (RpsF)	2.09
SP_0499	Phosphoglycerate kinase (Pgk)	2.06
SP_1799	Sugar-binding transcriptional regulator, LacI family	2.04
SP_0784	Glutathione reductase (Gor)	2.03
SP_0231	Adenylate kinase (Adk)	2.03
SP_1372	UPF0342 protein SP_1372	2.02
SP_2210	Cysteine synthase (CysM)	2.01

Only proteins with an increased fold change of 2 and above are shown

Supplementary Table 2

All proteins that iTRAQ analyses show have decreased expression in the MAV TIGR4.1 compared to the TIGR4 HKL preparation

SP #	Protein	Fold change
SP_0019	Adenylosuccinate synthetase (PurA)	-4.00
SP_0032	DNA polymerase I (PolA)	-2.58
SP_0035	Aromatic amino acid aminotransferase (AraT)	-2.10
SP_0071	Zinc metalloprotease ZmpC (ZmpC)	-2.51
SP_0085	30S ribosomal protein S4 (RpsD)	-2.56
SP_0095	UPF0176 protein	-3.09
SP_0103	Putative capsular polysaccharide biosynthesis	-5.17
SP_0117	Pneumococcal surface protein A (PspA)	-2.64
SP_0121	Metallo-beta-lactamase superfamily protein	-2.64
SP_0151	Methionine import ATP-binding protein MetN	-2.95
SP_0176	Riboflavin biosynthesis protein RibBA	-3.77
SP_0186	UvrABC system protein A (UvrA)	-2.80
SP_0202	Anaerobic ribonucleoside-triphosphate reductase (NrdD)	-2.59
SP_0208	30S ribosomal protein S10	-4.15
SP_0211	50S ribosomal protein L23 (RplW)	-2.31
SP_0212	50S ribosomal protein L2 (RplB)	-2.90
SP_0215	30S ribosomal protein S3	-4.99
SP_0216	50S ribosomal protein L16	-6.35
SP_0217	50S ribosomal protein L29 (RpmC)	-23.94
SP_0219	50S ribosomal protein L14 (RplN)	-2.67
SP_0220	50S ribosomal protein L24	-3.69

Supplementary Table 2 continued		
**SP #**	**Protein**	**Fold change**
SP_0221	50S ribosomal protein L5	-2.08
SP_0222	30S ribosomal protein S14 (RpsN)	-6.22
SP_0224	30S ribosomal protein S8	-4.16
SP_0225	50S ribosomal protein L6	-4.88
SP_0226	50S ribosomal protein L18 (RplR)	-2.64
SP_0228	50S ribosomal protein L30	-8.27
SP_0229	50S ribosomal protein L15	-4.51
SP_0232	Translation initiation factor IF-1	-14.61
SP_0234	30S ribosomal protein S13 (RpsM)	-2.09
SP_0264	Proline-tRNA ligase	-3.10
SP_0266	Glutamine--fructose-6-phosphate aminotransferase	-2.32
SP_0271	30S ribosomal protein S12	-4.30
SP_0294	50S ribosomal protein L13 (RplM)	-3.07
SP_0350	Capsular polysaccharide biosynthesis protein Cps4E	-3.73
SP_0355	Putative uncharacterized protein	-4.46
SP_0373	Putative uncharacterized protein	-2.30
SP_0413	Aspartokinase	-2.21
SP_0425	Acetyl-CoA carboxylase, biotin carboxylase (AccC)	-2.61
SP_0433	N utilization substance protein B homolog (NusB)	-2.43
SP_0439	Peptide chain release factor 3	-4.06
SP_0441	50S ribosomal protein L28	-3.80
SP_0443	Putative uncharacterized protein	-2.34
SP_0451	Putative uncharacterized protein	-2.92
SP_0493	Probable DNA-directed RNA polymerase δ (RpoE)	-5.46
SP_0494	CTP synthase (PyrG)	-2.76

Supplementary Table 2 continued		
**SP #**	**Protein**	**Fold change**
SP_0564	Putative uncharacterized protein	-2.72
SP_0613	Metallo-beta-lactamase superfamily protein	-2.17
SP_0630	50S ribosomal protein L11	-11.89
SP_0631	50S ribosomal protein L1	-7.14
SP_0648	Beta-galactosidase (BgaA)	-2.45
SP_0670	Putative uncharacterized protein	-4.17
SP_0675	Oxidoreductase, short chain dehydrogenase/reductase family	-2.80
SP_0678	Putative uncharacterized	-2.86
SP_0702	Orotate phosphoribosyltransferase (PyrE)	-2.57
SP_0752	Branched-chain amino acid ABC transporter, ATP-binding protein (LivG)	-2.65
SP_0756	Cell division ABC transporter, ATP-binding protein FtsE	-8.59
SP_0761	ATP-dependent RNA helicase, DEAD/DEAH box family	-2.87
SP_0762	S-adenosylmethionine (MetK)	-2.76
SP_0770	ABC transporter, ATP-binding protein	-3.08
SP_0780	Ribonucleoside-diphosphate reductase, alpha subunit, truncation	-2.83
SP_0788	Methionine-tRNA ligase	-3.87
SP_0801	Putative uncharacterized protein	-5.11
SP_0838	30S ribosomal protein S20	-13.86
SP_0846	Sugar ABC transporter, ATP-binding protein	-2.47
SP_0852	DNA topoisomerase 4 subunit B (ParE)	-2.36
SP_0855	DNA topoisomerase 4 subunit A (ParC)	-2.70
SP_0861	Putative uncharacterized protein	-2.62

Supplementary Table 2 continued		
SP #	Protein	Fold change
SP_0868	Putative uncharacterized protein	-2.65
SP_0871	Putative uncharacterized	-3.67
SP_0875	Lactose phosphotransferase system repressor	-5.18
SP_0876	Tagatose-6-phosphate kinase	-2.88
SP_0877	PTS system, fructose specific II ABC components	-3.59
SP_0897	Pyruvate kinase (Pyk)	-2.18
SP_0908	Putative transcriptional regulator	-3.82
SP_0923	Cof family protein	-2.04
SP_0929	Pseudouridine synthase (RluD)	-2.10
SP_0959	Translation initiation factor IF-3	-3.87
SP_0964	Dihydroorotate dehydrogenase B (NAD(+)), (PyrDB)	-6.16
SP_0969	GTPase Era	-2.36
SP_0985	Cys-tRNA(Pro)/Cys-tRNA(Cys) deacylase	-4.53
SP_1010	Large conductance mechanosensitive channel protein MscL	-2.35
SP_1025	Putative uncharacterized	-2.73
SP_1068	Phosphoenolpyruvate carboxylase (Ppc)	-2.83
SP_1087	ATP-dependent DNA helicase PcrA	-3.02
SP_1093	Putative uncharacterized protein	-5.41
SP_1095	Ribose-phosphate pyrophosphokinase 2 (Prs2)	-2.41
SP_1107	50S ribosomal protein L27	-4.56
SP_1128	Enolase	-2.75
SP_1155	Ribosome biogenesis GTPase A	-2.01
SP_1182	Lactose phosphotransferase system repressor (LacR)	-2.58
SP_1243	Glucose-6-phosphate 1-dehydrogenase (Zwf)	-2.14

Supplementary Table 2 continued		
**SP #**	**Protein**	**Fold change**
SP_1249	GMP reductase (GuaC)	-2.56
SP_1267	LicC protein	-2.36
SP_1269	Choline kinase (Pck)	-3.63
SP_1270	Alcohol dehydrogenase, zinc-containing	-5.43
SP_1275	Carbamoyl-phosphate synthase large chain	-4.89
SP_1276	Carbamoyl-phosphate synthase small chain	-4.96
SP_1278	Bifunctional protein PyrR	-2.61
SP_1293	50S ribosomal protein L19 (RplS)	-3.05
SP_1297	Flavodoxin (Fld)	-2.25
SP_1362	Adapter protein MecA (MecA)	-2.04
SP_1397	Phosphate import ATP-binding protein PstB 2	-2.51
SP_1421	Nicotinate phosphoribosyltransferase	-2.83
SP_1475	Glycine--tRNA ligase alpha subunit (GlyQ)	-2.19
SP_1483	ATP-dependent RNA helicase, DEAD/DEAH box family	-5.25
SP_1510	ATP synthase subunit alpha (AtpA)	-2.43
SP_1511	ATP synthase subunit delta (AtpH)	-2.14
SP_1521	UDP-N-acetylmuramate--L-alanine ligase (MurC)	-2.28
SP_1530	UDP-N-acetylmuramoyl-L-alanyl-D-glutamate--L-lysine ligase	-4.26
SP_1538	Cof family protein/peptidyl-prolyl cis-trans, cyclophilin	-2.92
SP_1539	30S ribosomal protein S18 (rpsR)	-2.31
SP_1542	Asparagine-tRNA ligase	-3.78
SP_1557	DegV domain-containing protein	-2.65
SP_1573	Lysozyme (LytC)	-7.32
SP_1580	Sugar ABC transporter, ATP-binding protein (MsmK)	-2.17

Supplementary Table 2 continued		
**SP #**	**Protein**	**Fold change**
SP_1584	GTP-sensing transcriptional pleiotropic repressor CodY	-4.23
SP_1586	Probable ATP-dependent RNA helicase (Exp9)	-2.36
SP_1631	Threonine-tRNA ligase (ThrS)	-2.02
SP_1667	Cell division protein FtsA	-3.04
SP_1674	Putative phosphosugar-binding transcriptional regulator	-2.54
SP_1701	Phospho-2-dehydro-3-deoxyheptonate aldolase	-2.54
SP_1702	Protein translocase subunit SecA 1	-3.05
SP_1737	DNA-directed RNA polymerase subunit omega (RpoZ)	-3.00
SP_1739	Ribonuclease Y	-4.23
SP_1749	GTP-binding protein	-2.40
SP_1837	Putative capsular polysaccharide biosynthesis protein	-2.96
SP_1853	Galactokinase	-10.13
SP_1880	Non-canonical purine NTP pyrophosphatase	-2.73
SP_1882	UPF0154 protein	-2.54
SP_1888	Oligopeptide transport ATP-binding protein (AmiE)	-2.18
SP_1894	Sucrose phosphorylase (GtfA)	-10.14
SP_1923	Pneumolysin (Ply)	-2.18
SP_1937	Autolysin	-10.98
SP_1940	Protein RecA	-2.36
SP_1960	DNA-directed RNA polymerase subunit β	-4.91
SP_1961	DNA-directed RNA polymerase subunit beta (RpoB)	-3.03
SP_1970	Aspartate-ammonia ligase (AsnA)	-2.50
SP_1975	Membrane protein insertase (YidC1)	-2.24
SP_1978	Diaminopimelate decarboxylase (LysA)	-3.04
SP_1979	Pur operon repressor	-7.99

Supplementary Table 2 continued		
**SP #**	**Protein**	**Fold change**
SP_1999	Catabolite control protein A (CcpA)	-2.62
SP_2030	Probable transketolase (Tkt)	-2.01
SP_2058	Queuine tRNA-ribosyltransferase (Tgt)	-2.80
SP_2076	DNA mismatch repair protein HexA	-2.31
SP_2078	Arginine-tRNA ligase (ArgS)	-2.11
SP_2121	Histidine--tRNA ligase	-4.39
SP_2135	50S ribosomal protein L33 type 3 (RpmG3)	-3.08
SP_2156	SPFH domain/Band 7 family	-2.81
SP_2190	Choline binding protein A (PspC)	-3.10
SP_2204	50S ribosomal protein L9	-4.24
SP_2230	ABC transporter, ATP-binding protein	-3.52

Only proteins with a decreased fold change of 2 and above are shown
